# Studying the unfolding process of protein G and protein L under physical property space

**DOI:** 10.1186/1471-2105-10-S1-S44

**Published:** 2009-01-30

**Authors:** Liling Zhao, Jihua Wang, Xianghua Dou, Zanxia Cao

**Affiliations:** 1 Key Lab of Biophysics in universities of Shandong (Dezhou University), Dezhou 253023, PR China; 2 Department of Physics, Dezhou University, Dezhou 253023, PR China

## Abstract

**Background:**

The studies on protein folding/unfolding indicate that the native state topology is an important determinant of protein folding mechanism. The folding/unfolding behaviors of proteins which have similar topologies have been studied under Cartesian space and the results indicate that some proteins share the similar folding/unfolding characters.

**Results:**

We construct physical property space with twelve different physical properties. By studying the unfolding process of the protein G and protein L under the property space, we find that the two proteins have the similar unfolding pathways that can be divided into three types and the one which with the umbrella-shape represents the preferred pathway. Moreover, the unfolding simulation time of the two proteins is different and protein L unfolding faster than protein G. Additionally, the distributing area of unfolded state ensemble of protein L is larger than that of protein G.

**Conclusion:**

Under the physical property space, the protein G and protein L have the similar folding/unfolding behaviors, which agree with the previous results obtained from the studies under Cartesian coordinate space. At the same time, some different unfolding properties can be detected easily, which can not be analyzed under Cartesian coordinate space.

## Background

Most proteins exist in unique three-dimensional conformations exquisitely suited to their function. Protein folding is one of the important and unsolved problems in life science. Some sophisticated theories have been proposed after several decades of extensive research through experimental and theoretical studies. The most popular theory is that native state topology is an important determinant of protein folding mechanism [1,2]. Studies on some small single-domain proteins suggest that proteins that have similar native structures with low sequence identity have similar transition state ensemble [3,4] and folding rates of two-state proteins have shown to correlate very well with contact order, a quality linked to topology [5,6]. On the other hand, there are also some exceptions. Some studies indicated that proteins with the similar native structures maybe have different folding pathway [7-9].

The above studies are usually performed under conformational space or geometrical space that is constructed based on the Cartesian coordinates of atoms. The three-dimensional structure of protein is changed during the folding/unfolding process. Companied with transformation of three-dimensional Cartesian coordinates of atoms, some physical parameters, such as native contact number, accessible surface area, radius of gyration, are correspondingly changed. Some parameters, such as the fraction of the native contacts Q[10], number of unfolded links μ, and the fraction of residues that are ordered N_*f *_[11], have been chosen as reaction coordinates to depict the protein folding/unfolding process. That is to say, physical property parameters representing some properties of protein can describe the characters during the process of protein folding/unfolding as the atoms three-dimensional Cartesian coordinates do. In this study, we investigate the protein folding/unfolding behaviors under physical property space which is based on physical property parameters. Some novel characters of protein folding/unfolding can be revealed under this physical property space [12].

**Figure 1:**
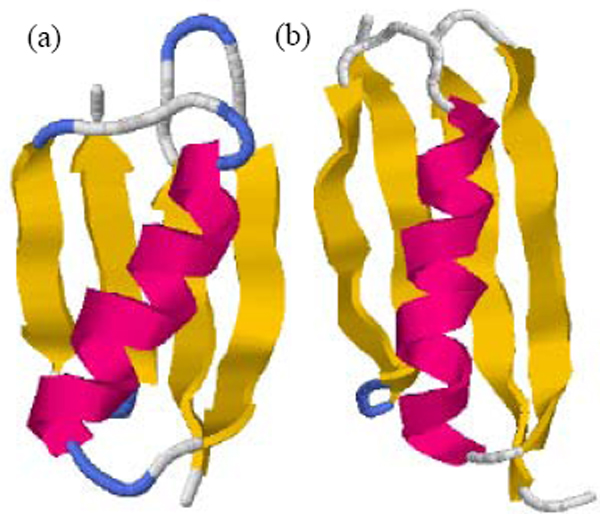
The structure of (a) protein G (2GB1 in PDB) and (b) protein L (2PTL in PDB).

The B1 IgG binding domain of streptococcal protein G (referred as protein G in this paper) and the B1 IgG binding domain of peptostreptococcal protein L (referred as protein L in this paper) share the similar native state topology. They both consist of a single α-helix packed against a four-stranded β-sheet formed by two symmetrically opposed β-hairpins (Fig. 1) [13-15] and have low homology (16% sequence identity). In this study, a physical property space was constructed based on twelve physical property parameters. Protein G and protein L were chosen as model systems to study the unfolding behaviors of proteins with similar native topology under the physical property space. Protein G has been studied by experiments and simulations as a model protein and possessed of many results to be compared. But protein L has not been studied by simulations as protein G widely. The results of protein L could be contrasted with that of protein G, as well as its former results. For each protein, forty independent thermal unfolding simulations were performed. With principal component analysis, the multidimensional physical property space had been reduced to three-dimensional essential property subspace. The unfolding trajectories and the unfolded state ensembles of the two proteins were studied under the essential subspace, and some results were different from those obtained under Cartesian coordinate space.

## Results

### Unfolding trajectory

**Figure 2:**
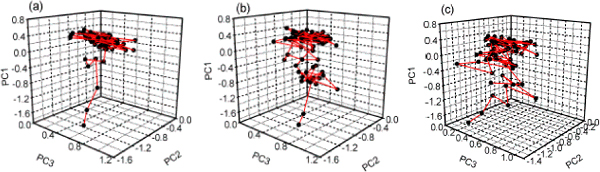
**The thermal unfolding trajectories of protein G under essential property subspace**. Eighty points were selected uniformly from twelve-nanosecond unfolded property trajectory to draw the figure. The forty unfolded property trajectories can be divided into three types; they were (a) type I, (b) type II and (c) type III, represented by trajectory 28, trajectory 12 and trajectory 29, respectively.

**Figure 3:**
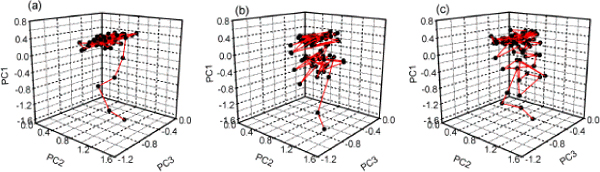
**The thermal unfolding trajectories of protein L under essential property subspace**. Eighty points were selected uniformly from twelve-nanosecond unfolded property trajectory to draw the figure. The forty unfolded property trajectories can be divided into three types; they were (a) type I, (b) type II and (c) type III, represented by trajectory 17, trajectory 14 and trajectory 35, respectively.

The unfolding trajectories obtained from the simulations with atoms coordinate of the two proteins were changed into unfolding property trajectories and projected into essential property subspace. The forty unfolding trajectories of each protein could be divided into three types in the subspace (Table 1). The trajectory number for type I, II and III were 22, 7, 11 for protein G and 22, 9, 9 for protein L, respectively. For the convenience of observation, only 80 points were selected uniformly from twelve-nanosecond trajectory to draw the figures (Fig. 2 and Fig. 3). One point represented one snapshot of protein during the simulations.

**Table 1:** The trajectory number of different unfolding types

Protein	type I	type II	type III
Protein G	22	7	11
Protein L	22	9	9

Type I had the same shape as umbrella (see Fig. 2(a) and Fig. 3(a)), and this type had 22 (55%) trajectories among forty unfolding trajectories for the two proteins. The probability of this type was much higher than that of the others.

### Unfolded state ensemble

When the native contact number in a conformation is less than 20% of that in the native state, the conformation was defined to be in the unfolded state ensemble [16]. Depended on the definition, fifty-five native contacts formed by fifty-six residues were identified for protein G, and forty-nine native contacts formed by sixty-two residues were identified for protein L.

**Figure 4:**
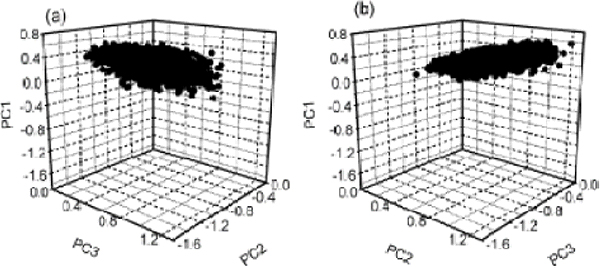
**The unfolded state ensemble of protein G and protein L under essential property subspace**. The unfolded state ensemble have the ellipsoid shape for (a) protein G and (b) protein L, represented typically by trajectory 28 and trajectory 17, respectively.

The Unfolded states in each trajectory were mapped into the essential property subspace. The forty trajectories had the similar unfolded state ensemble under the subspace for two proteins (Fig. 4). All the unfolded state assembles had the similar ellipsoid shape. The change ranges of the three principal components for unfolded state ensembles of two proteins were calculated (Table 2). The distributing area of unfolded state ensemble of protein L under the subspace was larger than that of protein G.

**Table 2:** The three principal components of unfolded states ensemble

Protein	Component	max	aver	min	range
Protein G	PC1	1.125	0.438	-0.127	1.252
	PC2	0.049	-0.662	-1.517	1.565
	PC3	0.735	0.250	-0.386	1.121
Protein L	PC1	0.240	-0.406	-1.146	1.386
	PC2	1.478	0.606	-0.145	1.622
	PC3	0.785	0.245	-0.491	1.276

### Unfolding simulation time

The time of the simulation before the protein unfolded into the unfolded state ensemble was called unfolding simulation time. The unfolding simulation time was employed to describe approximately unfolding rate for it is not the actual protein unfolding time. The actual unfolding time must be measured by experiment. The average unfolding simulation time of each unfolding type was calculated (Table 3). The average unfolding simulation time of protein L was approximately two thirds of that of protein G, but to type II, protein G unfolded approximately as faster as protein L. For protein G, the type II unfolded slower than type I and faster than type III. However, for protein L, the type II unfolded slower than type I and type III. Among the three unfolding types, type I unfolded fastest for both proteins.

**Table 3:** The average unfolding simulation time of each unfolding type (ps)

Protein	whole	type I	type II	type III
Protein G	2822	2064	3345	4004
Protein L	2134	1367	3391	2763

## Discussion

**Figure 5:**
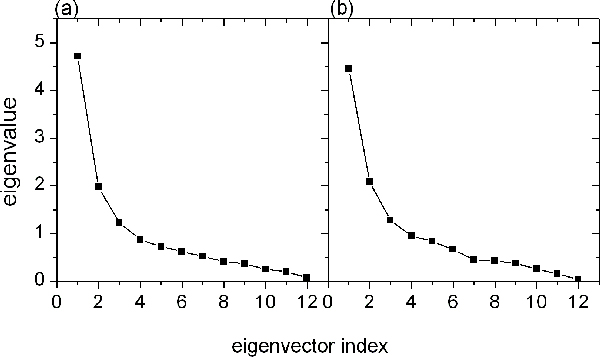
**The twelve eigenvalues of (a) protein G and (b) protein L**. The eigenvalues were obtained from average property covariance matrix in decreasing order of magnitude.

**Figure 6:**
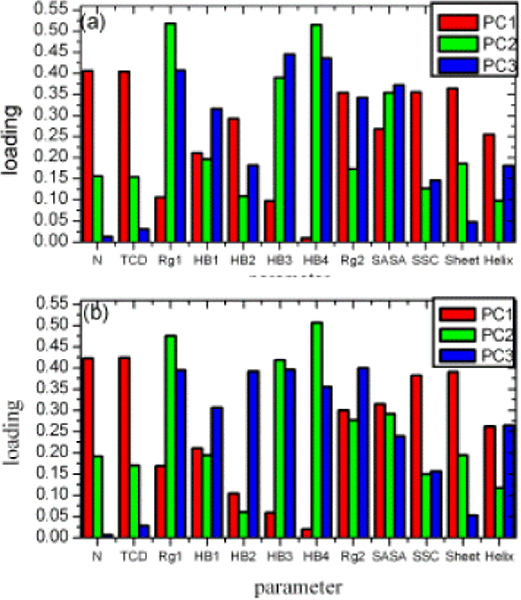
**The loadings of physical parameters for first three principal components of two proteins**. (a) protein G, (b) protein L. N is native contact number within the protein; TCD is total contact distance; Rg1 is radius of gyration of C_α _atom; HB1 is number of hydrogen bonds between the protein and waters within the hydrophobic core, HB2 is number of hydrogen bonds within the hydrophobic core, HB3 is number of hydrogen bonds within the protein, HB4 is number of hydrogen bonds between the protein and water, Rg2 is radius of gyration of the hydrophobic core, SASA is hydrophobic solvent-accessible surface area, SSC is second structure content, Sheet is the content of β sheet, Helix is the content of α helix.

One parameter can describe one property of protein. The more parameters were selected, the better conformation of the protein was represented. We selected twelve physical parameters [see Methods], which were often used in protein folding/unfolding analysis, to depict the character of protein at each time-step during the unfolding process. It was the changes of protein structure that were studied under Cartesian coordinate space. However, it was the changes of protein properties that were studied under physical property space. We could analyse the transformation of protein properties during the folding/unfolding process. Decreasing the twelve-dimension space to three-dimensional essential subspace could make the property trajectory be observed easily. The top three eigenvalues were much higher than the others [Fig. 5], which ensured that the subspace covered the main characters of twelve-dimension space. The different physical parameters has different loading for the three principal components for two proteins [Fig. 6]

With the protein spread, the native contact number N decreased, at same time, the total contact distance TCD became smaller and the second structure content SSC including the β sheet content decreased. For the inverse proportion with those parameters, the first principal component PC1 increased accordingly (Fig. 2, 3). The second principal component PC2 was dominated by the loading of radius of gyration of C_α _atom Rg1 and number of hydrogen bonds between the protein and water HB4. During the unfolding process, the protein unwound and its volume became bigger, and the more hydrogen bonds between the protein and water were formed, then the second principal component PC2 was increased in physical property space until the unfolding simulation convergence. The variety of PC3 was the same as PC1 and PC2.

Under the essential property subspace, the unfolding trajectories of protein G and protein L had the similar three types, which was coordinate with the fact that native state topology determines the folding mechanism. Type I with umbrella-shape had the higher appearance probability and the shortest unfolded simulation time among the three types, which might induced that this type was the preferred pathway among the multiple pathway [17]. The shape of unfolding trajectory of type I indicated that the two proteins were fast folding two-state proteins, which was consistent with the fact that protein G and protein L are fast folding two-state kinetics proteins [18,19]. For the similar topology, protein G and protein L had the similar unfolded state ensemble as ellipsoid.

Protein G and protein L have low homology (16% sequence identity) although they share the similar native state topology. The most obvious difference for the two proteins is the α-helix orientation. In protein L the helix is almost parallel to the β-sheet, whereas in the protein G the helix runs diagonally across the sheet [15]. Studies indicate that one of the two β-turns is largely formed and the other largely disrupted in the folding transition state. In protein L it is the N-terminal β-turn, and in the protein G the C-terminal β-turn, that is formed in the transition ensemble [7-9,20]. However, the difference disappeared under the property space for the two proteins. The property space was constructed by physical property parameters of protein, for the two proteins, different transient states in Cartesian space might have the same physical properties. At same time, some other difference of unfolding behaviors of the two proteins was observed obviously under the property space.

First, the unfolding simulation time was difference. Protein L unfolded much faster than protein G, especially for the type I which represented the preferred pathway, which was accordant with the experiments study [19,21]. It may be related to the amino acid sequence of the two proteins. Protein G and protein L had different residue number and different native contact number though they had the similar native topology structures. The ratio of local contact among all native contacts was different between protein G and protein L [7,8] and the local and non-local contacts had different influence on unfolding rate.

Second, the distributing area of unfolded state ensemble was different. The unfolded states resided in a finite area under the property space, which agreed with the fact that the unfolded states are not infinite but finite [22,23]. For protein L, the change ranges of three principal components were larger than those of protein G, and corresponding distributing area of unfolded state ensemble of protein L was larger than that of protein G. With similar native topology, protein L had the more unfolded states.

Among the multiple folding pathways, the unfolding difficulty of protein was different. Protein L had more unfolding difficulty by type II than by the other two types and protein G had middle unfolding difficulty by type II among the three unfolding types. For the same reason, protein L unfolded slower than protein G by type II.

The closer of the two points were in property subspace, the more similar properties the two conformations had. For type II, proteins unfolded from native state to unfolded state with an obvious stagnation at some states. There might be a intermediate state for the two proteins, which required further study to be confirmed.

In the physical property space, a point represents a conformation of protein that have thousands of atom coordinates in Cartesian space. The whole behaviors of protein folding/unfolding can be observed easily. With some effective analysis tool such as network, some details of protein folding mechanism may be detected, which is worthy of the following study.

## Conclusion

In this study, the physical property space was constructed with twelve physical parameters and decreased to three-dimensional essential property subspace. Under the property space, the unfolding behaviors of protein G and protein L were studied. With the statistical analysis on the forty unfolding property trajectories, we found that the two proteins with similar native state topologies had the similar unfolding property trajectories and similar unfolded state ensemble under the property space, which agreed with the previous study under Cartesian space. At the same time, some unfolding properties, which could not be realized by studies under Cartesian space, could be easily detected, for example, the unfolding pathway type, the difference of unfolding simulation time and the difference of distributing area of the unfolded state ensemble. At last, we only studied the two proteins and can not say that all proteins with similar native topology have the same characteristic under property space as protein G and protein L, which demands more deep research.

## Methods

### Molecular dynamics (MD) simulations

The initial conformations of protein G and protein L were taken from protein data bank (PDB) [24] with PDB entry code 2GB1 and 2PTL, respectively, which have been solved by NMR spectroscopy [13-15]. Unfolding simulations were carried out using the GROMACS software package [25] with the GROMOS96 43a1 force field [26] and explicit water. The SPC water model was used for water molecules [27]. After energy minimization, some water molecules were replaced with same number of chlorine or natrium ions to neutralize the system. Under 300 K and 1 bar, position-restrained MD simulations were performed for 500 ps and forty conformations were received every 10 ps from the last 400 ps simulation trajectory. With each conformation, position-restrained MD simulations were performed for 100 ps under 540 K and 1 bar at first, and then free MD simulations were carried out for 12ns under same condition. The time step of simulation is 2 fs and the total simulation time is up to 0.96μs.

### Physical property

For the convenience of analysis, 6000 conformations were chosen for the following analysis from 12 ns unfolding trajectories with same interval. The following twelve physical properties were calculated, they are α helix content, β sheet content, second structure content (including α helix, β sheet, β bridge, bend and turn), hydrophobic solvent-accessible surface area, radius of gyration of C_α _atom, number of hydrogen bond within the protein, radius of gyration of the hydrophobic core, number of hydrogen bond within the hydrophobic core, number of hydrogen bond between the protein and water, number of hydrogen bond between the protein and waters within the hydrophobic core, native contact number within the protein, and total contact distance (TCD) [20].

We define a contact as being present if the C_α _atom of two residues (i, j) are within 6.5 angstrom. We define native contact including all contact formed between residues not adjacent in sequence and present in both reference native simulations for more than two-thirds of the simulation time [16].

### Principal component analysis and property space

The twelve parameters mentioned above of protein were calculated for each conformation during the simulations. The value of each parameter was normalized between 0 and 1, with 0 corresponding to the lowest value across the trajectory and 1 being the highest value across the trajectory. The covariance property matrix *C *was calculated and the element *c*_*ij *_was determined by

*c*_*ij *_= ⟨(*x*_*i *_- ⟨*x*_*i*_⟩)(*x*_*j *_- ⟨*x*_*j*_⟩)⟩

where ⟨⟩ donated the average over all structures sampled in the trajectory and *x*_*i *_= *x*_*i*_(*t*) was the *i*^th ^physical parameter of the conformation at time *t*.

The property matrix *C *was 12 × 12 dimensional symmetric matrix. An average property matrix was obtained by averaging forty property matrixes. With the principal component analysis, the average property matrix was diagonalized to get the twelve new orthogonal eigenvectors and corresponding eigenvalues. The first three eigenvectors with largest eigenvalues were selected as three principal components $\vec{PC_{1}}$, $\vec{PC_{2}}$, $\vec{PC_{3}}$ to construct three-dimensional essential physical property subspace, and the unfolding trajectories were projected into the subspace.

## Competing interests

The authors declare that they have no competing interests.

## Authors' contributions

Liling Zhao carried out most of the theoretical analysis and drafted the manuscript, while Jihua Wang carried out some of the theoretical analyses and designed the research project and Zanxia Cao revised the paper. Xianghua Dou and Liling Zhao performed the molecular dynamics simulations. All authors read and approved the final manuscript.
